# Blood buffers: The viewpoint of a biochemist

**DOI:** 10.14814/phy2.70333

**Published:** 2025-05-05

**Authors:** Andrea Bellelli

**Affiliations:** ^1^ Department of Biochemical Sciences “A. Rossi Fanelli” Sapienza University of Rome Rome Italy

**Keywords:** acidosis, alkalosis, CO_2_, hypercapnia, hypocapnia, SID

## Abstract

Mammalian blood is a very complex system whose multiple physiological roles require that its pH is maintained constant, in spite of the necessity of carrying over 15 moles of CO_2_ a day from the tissues to the lungs. The blood pH is maintained constant by several buffers, whose interplay is complex. The study of blood buffers is over a century old and has crossed major reinterpretations of the nature of acids and bases, from Arrhenius to Bronsted and Lowry, as well as an enormous evolution of our knowledge of protein structure, proteins being the most relevant among blood buffers. As a consequence, several theories have been developed to explain the physiological and pathological fluctuations of blood pH. This review compares the three main theories currently used: that based on the Strong Ions Difference (SID), due to Stewart and his followers; that based on the Base Excess, due to the Copenhagen school of respiration physiology; and the physiological one, due to the Boston school. These theories are not alternative but complementary and can be reconciled with each other, provided that some erroneous assumptions are corrected, and all three are expressed using the definitions proposed by Bronsted and Lowry.

## 1 INTRODUCTION

The buffer capacity of blood in vitro is mainly due to the titratable amino acid residues on the surface of blood proteins, preeminently of hemoglobin, in view of its concentration. The CO_2_/bicarbonate system contributes to a greater extent in vivo than in vitro because of its continuous production and excretion. A huge amount of information has been gathered on this subject, but the interpretation of the acid base chemistry of blood is still debated for several reasons: (i) the intrinsic complexity of blood and its exchanges with extracellular and intracellular fluid; (ii) the fact that the problem has been attacked since over one century during which our understanding of acid and bases, protein chemistry and organ functions has changed in very significant ways, leading to the stratification of clinically useful concepts that are scarcely compatible with each other; (iii) the fact that only some of the relevant parameters are accessible to direct measure, all the others being derived from theoretical and empirical considerations that may not grasp the full picture. The aim of this review is to bridge some of the gaps between these different approaches, and try to make the whole issue less “confusing, irrational and controversial”, to quote a definition by Berend (2013). In some cases, it is necessary to go back in time and review very old data. In this review I shall mainly focus on physiological mechanisms, and shall not enter the very specialized field of diagnosis of acid–base disorders.

## 2 THE BUFFER CAPACITY OF BLOOD IN VITRO

Homeostasis of the pH of body fluids is essential for life, and relatively small deviations from the values observed in healthy subjects entail significant risk. Since blood is a very complex medium, the complete definition of the buffers it contains is difficult, also because assessment of its acid balance status only relies on a few easily measurable parameters: pH, bicarbonate concentration, the pressure of CO_2_ in equilibrium with blood (PCO_2_), overall buffer capacity, the anion gap, the standard base excess, and a few others. Some of these are correlated by simple mathematical equations; for example, pH, bicarbonate, and PCO_2_ are related to each other by the Henderson‐Hasselbalch equation, thus measuring only two is enough, and the third can be calculated.

**FIGURE 1:**
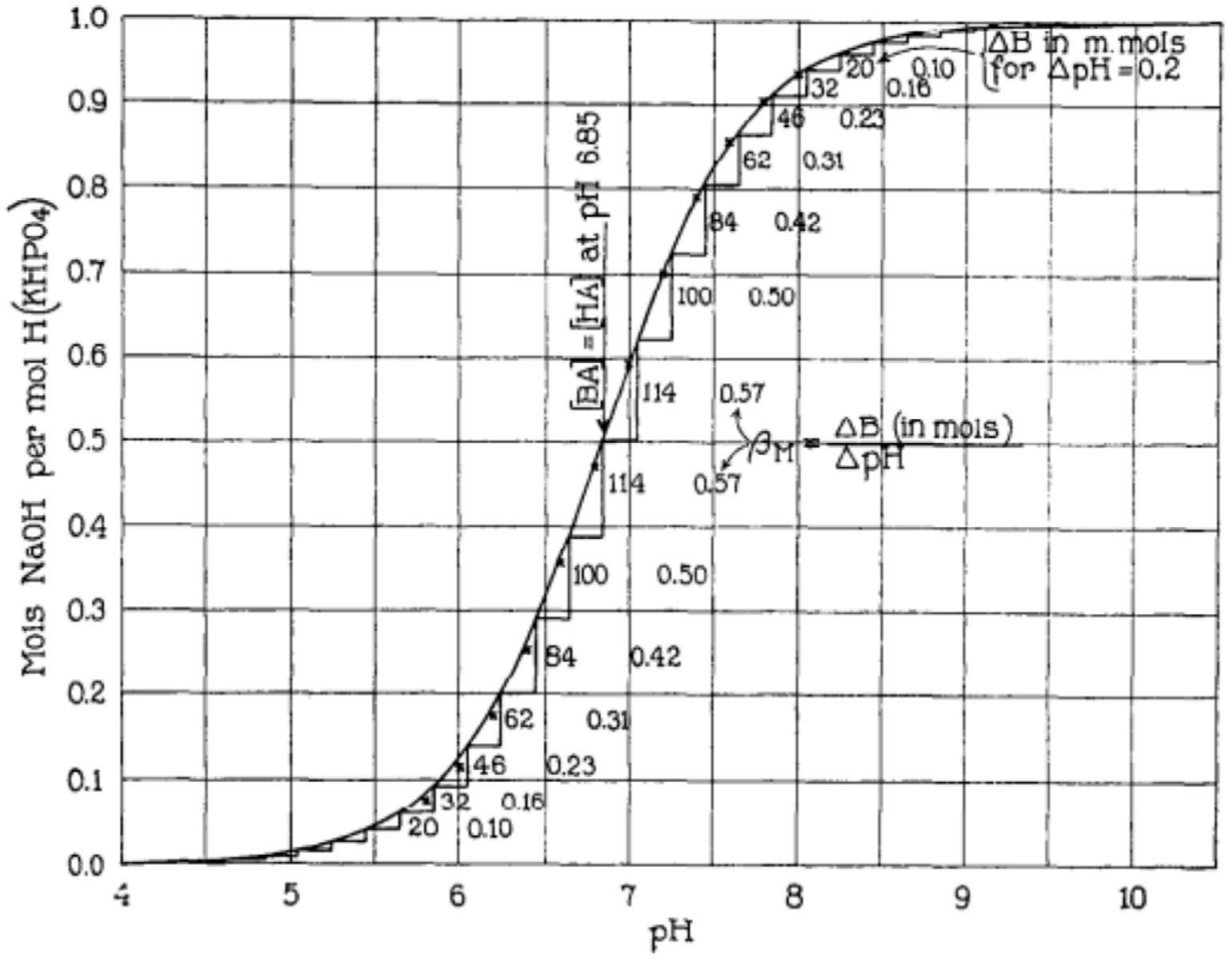
Buffer capacity of KH_2_PO_4_ titrated with NaOH. Reproduced from Van Slyke (1922) (copyright expired). Thanks are due to the American Society of Biochemistry and Molecular Biology for making this content available.

The blood contains several buffers, each having its own pK_a_ and its own base to acid ratio. The most important buffer, whose concentration exceeds bicarbonate by a significant margin, is provided by the titratable amino acid residues on the surface of proteins, most notably hemoglobin. The state of protein buffers is difficult to quantify, except via their contribution to the buffer capacity of blood. The concept of buffer capacity (also called β, buffer power, or buffer value) was defined by Van Slyke (1922) as the derivative of the titration curve of the buffer (see Figure 1). The buffer capacity is measured in moles of strong acid or base per mole of buffer per unit of pH change or, when the buffer concentration is unknown, in moles of strong acid or base per liter of solution per unit of pH change (mEq/L ΔpH). The relevance of this concept for the analysis of a complex mixture of buffers is that the buffer capacity can be measured empirically and is additive, that is, it estimates the sum of the contribution of all buffers at a given pH, even under conditions where one does not know which buffers are present, at which concentration and with which pK_a_.

The buffer capacity of whole human blood and its separated components has been measured by several authors over time; the interindividual variability is relatively large, but a reasonable average estimate is as follows: whole blood 31–38 mEq/L; plasma 16 mEq/L (Ellison et al., 1958). Assuming a hematocrit of 40%, the buffer capacity of whole blood may be partitioned as follows: plasma ≈25%; red blood cells ≈75%. The major component of the buffer capacity of erythrocytes is hemoglobin, which may be expected to account for approximately 65% of the total buffer capacity of blood, but bicarbonate and organic and inorganic phosphates also play a role.

The buffer capacity of plasma is due to several components that include proteins, bicarbonate, and phosphate. The contribution of bicarbonate can be estimated from its physiological concentration to be approximately one fourth of the buffer capacity of plasma (4 mEq/L), but it increases in conditions of acidosis, as the blood pH becomes closer to the pK_a_ of this buffer (pK = 6.1 at T = 37°C) (Ellison et al., 1958). I remark in passing that the pK_a_ of CO_2_ is defined for the reaction: CO_2_ + 2 H_2_O ⇋ HCO_3_^−^ + H_3_O^+^ and results from the sum of the pK of hydration of CO_2_ to H_2_CO_3_ and the pK_a_ of the first acid dissociation of the latter. Plasma proteins, organic and inorganic phosphate, and other components account for the remaining fraction of the buffer capacity of plasma (Constable, 1997). Non‐carbonic buffers of plasma and whole blood are not measured directly in the blood gas analysis and behave as “hidden buffers” of which only the overall buffer capacity can be estimated. It is important to remember that the concentration of bicarbonate measured in the plasma (average value in healthy subjects 24–26 mM) is higher than in whole blood (20.5–22.5 mM) because of the lower anion concentration in the erythrocytes.

In spite of its relatively small contribution to the buffer capacity of blood, CO_2_ is produced and excreted at a very high rate; thus, imbalances in its excretion may exert huge effects on the blood pH. The amount of CO_2_ eliminated by respiration (8–10 mmoles/min. or 1.6–2 mmoles per liter of blood passing through the pulmonary capillaries) exceeds the blood concentration of the gas (1.1–1.3 mM). In order to sustain the respiratory elimination of CO_2_, bicarbonate must be converted into the gas form by carbonic anhydrase, and this reaction requires proton donation by the hidden buffers:

(1) $${{\mathrm{HCO}}_{3}}^{-}+{\mathrm{BH}}^{+}⇋H_{2}{\mathrm{CO}}_{3}+B⇋{\mathrm{CO}}_{2}+H_{2}O+B$$

Thanks to the hidden buffers and mainly to hemoglobin, the pH increase in the pulmonary capillaries is less than 0.1 pH units.

## 3 THE BUFFER CAPACITY OF THE HIDDEN BUFFERS

The most important hidden buffers in blood, ordered according to their relevance, are hemoglobin, serum albumin, and organic and inorganic phosphates. The concentration of hemoglobin in the blood of a healthy adult ranges between 13 and 15 g/dL, corresponding to approx. 2.2 mmoles of tetramer/L of blood. The most important amino acid residues responsible for the buffer capacity of hemoglobin are histidines, given that their pK_a_ is closest to the physiological pH; other residues may contribute to a lesser extent (e.g., the α‐amino group of the N‐terminal valines of the α subunit). Every tetrameric molecule contains 38 His residues (10 in the α subunit, and 9 in the β subunit (Guidotti et al., 1962)) and this alone provides an estimated buffer concentration of ≈80 mEq/L. Table 1 reports the pK_a_ values and contribution to buffer capacity of the main buffer residues of hemoglobin. Note that not all His residues have been assigned.

**TABLE 1:** Buffer capacities of hemoglobin by selected amino acid residues and the other blood buffers.

Residue	pK_a_ HbO_2_	pK_a_ Hb	HbO_2_ buffer capacity	Hb buffer capacity
Val α1	7.25	8	2.46	1.62
His α20	7.08	7.02	2.22	2.10
His α45	6.12	Not determined	0.48	
His α50	6.9	7.14	1.85	2.32
His α72	7.27	7.47	2.47	2.51
His α89	6.25	6.8	0.62	1.62
His α112	7.53	7.49	2.47	2.50
His β2	6.39	6.17	0.82	0.53
His β77	7.79	7.46	2.08	2.52
His β97	7.75	8.01	2.16	1.60
His β116	6.13	6.35	0.49	0.76
His β117	6.39	6.43	0.82	0.88
His β143	5.57	4.7	0.15	0.02
His β146	6.42	7.93	0.87	1.78
Total assigned buffer capacity of hemoglobin (mEq/L of blood)			17.5	19.2

Other buffers (per liter of whole blood)		
Buffer	Concentration	Buffer capacity
Total buffer capacity of serum albumin	7 mEq/L	2.3 mEq/L
Buffer capacity of CO_2_/bicarbonate	22 mMol/L	2.5 mEq/L
Total buffer capacity of phosphates	6 mEq/L	2.8 mEq/L
Unassigned buffer capacity		5–10 mEq/L

*Note*: pK_a_ values of His residues of hemoglobin from Fang et al. (1999); of Val α1 from Perutz et al. (1980). The concentration of hemoglobin is assumed to be 2.2 moles of tetramer/L of blood, which makes the concentration of each of the listed residues 4.4 mM; the buffer capacity is calculated at pH = 7.4, 0.1 M chloride, T = 25–29°C. Note that, since all the residues are weak bases, their pK_a_ values are given for the hydrolysis reaction, BH^+^ + H_2_O ⇋ B + H_3_O^+^, that is, as pK_a_ = pK_w_ − pK_b_. Data for serum albumin from Watson (1999). Buffer capacity (in mEq/L of whole blood) was calculated using the equation: *β* = 2.3 × K_a_ × C × [H_3_O^+^]/(K_a_ + [H_3_O^+^])^2^ (Van Slyke, 1922).

**FIGURE 2:**
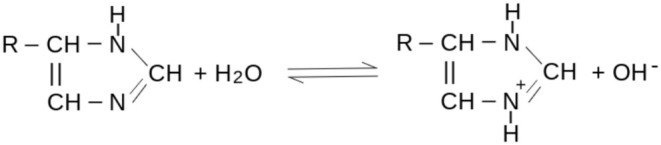
The acid–base reaction of His, whose pK is relevant to blood buffers (see Table 1).

The total buffer capacity assigned to hemoglobin by the values reported in Table 1 (17.5–19.2 mEq/L of blood or 8–9 mEq/mmol of tetramer) is slightly lower than the value estimated from direct titration of blood (20–24 mEq/L, see above); this discrepancy may be attributed to the contribution of other residues on the surface of the macromolecule. Buffer capacity is poorly related to the net charge of the protein that at pH = 7.4 is negative due to Asp and Glu residues, whose pK_a_ is too low to act as buffers. By contrast, His residues are neutral or positively charged (Figure 2).

Other unequivocally identified hidden buffers are the His residues of serum proteins, notably of albumin, and organic and inorganic phosphates (mostly present in the red cell cytoplasm). Their estimated contribution to the buffer capacity of blood is also reported in Table 1. Notice that the measured buffer capacity exceeds by 5–10 mEq/L the buffer capacity we can account for, presumably because of the contribution of other proteins and low molecular weight components (e.g., globulins, membrane proteins, low molecular weight peptides, etc.).

## 4 A SUMMARY OF THE CONCEPTS USED TO DESCRIBE THE ACID–BASE PROPERTIES OF BLOOD

In a typical gas analysis of arterial blood, the following parameters are measured or derived from calculations: partial pressure of CO_2_ (PCO_2_), pH, serum bicarbonate concentration, base excess (or standard base excess, see below), and serum anion gap (the difference between measured cations and measured anions, mostly due to protein anions). PO_2_ is also determined, but it does not provide direct information on the acid–base equilibria, even though it may (or may not) correlate with alterations of PCO_2_.

Alterations of blood pH are called acidoses and alkaloses, and may be due to respiratory or “metabolic” (non‐respiratory) causes. Respiratory effects occur because of physiological or pathological changes in the efficiency of CO_2_ exchange. Metabolic conditions include accumulation or loss of acid metabolites different from CO_2_, as well as conditions in which the renal excretion of bicarbonate and other acidic or basic substances is impaired. The excretory functions of the lung and kidney regulate the pH of all body fluids; when either of them is impaired, the other tries to compensate. Discriminating between pathological and compensatory effects is not always easy.

Three main approaches, each with some variants, have been devised to describe the acid–base balance of blood: (i) the physico‐chemical description; (ii) the measurement of other, less obvious parameters (e.g., the standard bicarbonate or the base excess); and (iii) the so‐called physiological, or bicarbonate‐based, approach.

## 5 THE PHYSICO‐CHEMICAL MODEL AND THE (WRONG) CONCEPT OF BUFFER BASE, OR STRONG IONS DIFFERENCE

Early attempts to describe the acid–base balance of blood under normal and pathological conditions relied on the determination of total CO_2_ (i.e., the sum of CO_2_, bicarbonate and protein carbamates). Indeed, in the first decades of the XX century, an increase in total CO_2_ was assumed to represent alkalosis, while a decrease in total CO_2_ was assumed to represent acidosis (Singer & Hastings, 1948). Van Slyke warned that exceptions to this rule, which assumed normal respiratory function, are possible, but the most relevant advance over this formula is due to Singer and Hastings, who stressed the necessity of additional parameters for a full assessment of the acid–base status of blood (Singer & Hastings, 1948). Singer and Hastings, as all previous authors, adopted a modified version of Arrhenius' definition of acids and bases (Story, 2004), and considered chloride and other anions as acids and sodium and other cations as bases; that is, they considered Cl^−^ as derived from the dissociation of HCl and thus either associated to a proton or neutralized by a base (e.g., NaOH, as in NaCl). This approach had already been made obsolete by Bronsted and Lowry's theory of acids and bases, but was still current in medical texts; hopefully, the two theories of Arrhenius and Bronsted and Lowry can be translated into each other (with some effort), but their coexistence was, and still is, a reason for confusion. Anions that do not react with water or recombine with hydrogen ions (e.g., chloride) are called “fixed acids” or “strong anions” On the contrary, anions that react with water and recombine with hydrogen ions (e.g., bicarbonate) are called “buffer anions”; protein anions were assumed to belong to this category, but this was an error because the main amino acid residues that confer buffer capacity to blood proteins are cations, rather than anions (Figure 2, Table 1). Singer and Hastings assumed cations to be essentially fixed cations, which they called “total bases” (B^+^). Total bases are conceptually divided into two fractions: the counterions of buffer anions (buffer bases, BB^+^) and the counterions of fixed acids (B^+^ − BB^+^). If one measures the concentration of total bases in the serum (essentially the sum of metal cations, dominated by sodium concentration) and that of fixed acids (dominated by chloride), one may obtain an approximate estimate of the buffer bases: [BB^+^] = [Na^+^] − [Cl^−^]. The erroneous concept of buffer base is a fundamental parameter in Singer and Hastings' theory of blood acid–base properties, as it is supposed to monitor the metabolic component of blood buffers; it is complemented by the other fundamental parameter PCO_2_, which monitors the respiratory component of blood buffers.

In the late 1970s, Peter Stewart proposed a model to describe the acid–base equilibrium of blood that mixes some excellent premises with some incorrect assumptions (Stewart, 1978); this model can be considered an evolution of the one proposed by Singer and Hastings. Unsurprisingly, Stewart's model gained some admirers and many critics.

Stewart's model assumes (correctly) that one can simulate the acid–base properties of blood by postulating the presence of only two buffers: CO_2_ and a weak acid of known pK_a_. This drastic simplification holds only as long as the pH interval of interest is small; but this limit is acceptable given that life is compatible only with a small blood pH interval. The independent parameters of Stewart's model are: PCO_2_; the total concentration of the non‐carbonic buffer (C_tot_) with its apparent pK_a_; and the strong ions difference (SID, fixed cations minus fixed anions, equivalent to BB^+^), whose role is to represent the sum of the deprotonated components from the two buffers. All other conceivable parameters (pH, [HCO_3_^−^], etc.) are dependent parameters. The distinction of dependent and independent parameters appears somewhat arbitrary (Kurtz et al., 2008), but should be interpreted in the sense that independent parameters must be imposed upon the model, whereas dependent parameters can be calculated by the model. Stewart's equations are complicated because he avoided simplifications, ending up with fourth‐degree equations. The model can be simplified without loss of information or predictive ability, as shown by Constable (Constable, 1997), who considered only three chemical reactions:

(2) $${\mathrm{CO}}_{2}+2\;H_{2}O⇋{{\mathrm{HCO}}_{3}}^{-}+H_{3}O^{+}$$

(3) $$\mathrm{AH}+H_{2}O⇋A^{-}+H_{3}O^{+}$$

(4) $${\mathrm{CO}}_{2}+A^{-}+H_{2}O⇋\mathrm{AH}+{{\mathrm{HCO}}_{3}}^{-}$$

The last equation expresses the conservation of charge and tells us that in this system the sum of the deprotonated components of the two buffers is constant, as was anticipated by previous authors (Van Slyke, 1922). Since in the model the non‐carbonic buffer is a weak acid (AH) and because of electroneutrality, the sum [A^−^] + [HCO_3_^−^] corresponds to SID (or BB^+^). The reader will notice that Equation 4 is identical to Equation 1, except that Equation 1 more realistically considers the non‐carbonic buffer a weak base. Stewart did not consider the possibility of basic buffers, and this error has important consequences. The existence, and actually the preeminence, of cationic buffers in blood was noticed by several authors (Constable, 1997; Figge et al., 1991, 1992; Watson, 1999), who tried to circumvent the problem, as I shall discuss later on.

The great merit of Stewart's approach is that his model can be formalized by a rigorous physico‐chemical description: indeed from Equations (2), (3), (4) we derive:

(5) $$C_{\mathrm{tot}}=\left[A^{-}\right]+\left[\mathrm{AH}\right]$$

(6) $$\mathrm{SID}=\left[A^{-}\right]+\left[{{\mathrm{HCO}}_{3}}^{-}\right]$$

(7) $$\left[H_{3}O^{+}\right]=K_{c}\;0.03\;{\mathrm{PCO}}_{2}/\left[{{\mathrm{HCO}}_{3}}^{-}\right]=K_{a}\;\left[\mathrm{AH}\right]/\left[A^{-}\right]$$

where K_c_ is the equilibrium constant of the dissociation of CO_2_ and K_a_ that of the weak acid AH. The model has three variables (PCO_2_, C_tot_, and SID), and three constants (the solubility coefficient of CO_2_, K_a_, and K_c_). SID can be easily determined from a clinical laboratory analysis of blood electrolytes, and its value under physiological conditions is SID ≈ [Na^+^] − [Cl^−^] ≈35–40 mEq/L. Strictly speaking, C_tot_ and K_a_ are unknown because the non‐carbonic buffer is an abstraction that collects many different buffers, but these parameters can be guessed from the buffer capacity of the system.

From Equations (5), (6), (7) we derive:

(8) $$K_{c}\;0.03\;{\mathrm{PCO}}_{2}/\left(\mathrm{SID}-\left[A^{-}\right]\right)=K_{a}\;\left(C_{\mathrm{tot}}-\left[A^{-}\right]\right)/\left[A^{-}\right]$$

(9) $${\left[A^{-}\right]}^{2}-\left[A^{-}\right]\;\left(\mathrm{SID}+C_{\mathrm{tot}}+0.03\;{\mathrm{PCO}}_{2}\;K_{c}/K_{a}\right)+\mathrm{SID}\;C_{\mathrm{tot}}=0$$

Once [A^−^] has been found, it is a trivial matter to calculate all the dependent variables of the model: [H_3_O^+^], pH, [AH], and [HCO_3_^−^].

While Stewart's model has great explanatory power from a theoretical point of view, its application to the blood fails to predict some important features, for example, it severely underestimates the buffer capacity. As already stated, the main non‐carbonic buffers of blood are positively charged His residues of proteins, and this completely nullifies the concepts and significance of buffer anions and SID. Indeed, SID is only slightly higher than bicarbonate alone and underestimates the hidden buffers (see Table 2). Moreover, the contribution of hemoglobin, which is intracellular, is completely unrelated to SID, which is measured in the plasma.

**TABLE 2:** CO_2_ excretion by the lung as modeled using Equation 10, compared with the SID‐based Equation 9.

	Healthy arterial blood, this work	Healthy venous blood, this work	Healthy arterial serum (Constable, 1997)	Healthy venous serum (Constable, 1997)
Non‐C buffer (C_tot_)	77 mEq/L	77 mEq/L	20 mEq/L	20 mEq/L
pK_a_	7	7	6.5	6.5
PCO_2_	38 mmHg	45 mmHg	38 mmHg	45 mmHg
pK_CO2_	6.1	6.1	6.1	6.1
S_dp_/SID	79 mEq/L	79 mEq/L	40 mEq/L	40 mEq/L
Buffer capacity (β)	38 mEq/L	40 mEq/L	7.1 mEq/L	8.2 mEq/L
[A^−^]	55.5 mEq/L	54 mEq/L	17.7 mEq/L	17.4 mEq/L
[HA]	21.5 mEq/L	23 mEq/L	2.28 mEq/L	2.61 mEq/L
[HCO_3_^−^]	23.5 mM	25.1 mM	22.3 mM	22.6 mM
Total CO_2_	24.6 mM	26.4 mM	23.4 mM	24.0 mM
Arterio‐venous CO_2_ difference	1.8 mM		0.54 mM	
pH	7.41	7.37	7.39	7.32

*Note*: The values one would obtain using Constable's parameters for human serum (Constable, 1997) are reported for comparison. Notice that the approach described in this work makes use of the parameter S_dp_, (columns 2 and 3) whereas that by Stewart and Constable makes use of SID (columns 4 and 5; see text).

A clever, but still unsatisfactory, solution to this problem was envisaged by Fencl and co‐workers (Figge et al., 1991, 1992); see also (Watson, 1999). These authors, working on plasma rather than on whole blood, recognized the problem of cationic buffers and identified three main buffers: bicarbonate, serum albumin, and phosphate. This yields:

$$\mathrm{SID}=\left[{{\mathrm{HCO}}_{3}}^{-}\right]+\left[\text{albumin charge}\right]+\left[\text{phosphate charge}\right].$$

The net charge of albumin is contributed by 99 fixed anions (Glu and Asp residues, which are deprotonated at physiological pH), 77 fixed cations (Lys and Arg) and a fraction of the 16 cations potentially provided by His residues, which play the major role as buffers. Thus, the negative charge assigned to albumin is:

$$\text{Negative charge of albumin}\;\left(\mathrm{mEq}/L\right)=\left[\text{albumin}\right]\;\left(22-16\;\left[H_{3}O^{+}\right]/\left(K_{a}+\left[H_{3}O^{+}\right]\right)\right)$$

This formula correlates the negative charge of albumin to the protonated/deprotonated state of its His residues. Unfortunately, this solution cannot account for hemoglobin. Moreover, if one makes use of the measured values of pH and the concentrations of albumin and bicarbonate, there is no need at all to use SID.

For a radical correction, SID should be replaced in Equation 9 with the concept “sum of the deprotonated buffer components”, which I shall indicate as S_dp_:

$$S_{\mathrm{dp}}=\left[B\right]+\left[{{\mathrm{HCO}}_{3}}^{-}\right]$$

(10) $${\left[B\right]}^{2}-\left[B\right]\;\left(S_{\mathrm{dp}}+C_{\mathrm{tot}}+0.03\;{\mathrm{PCO}}_{2}\;K_{c}/K_{a}\right)+S_{\mathrm{dp}}\;C_{\mathrm{tot}}=0$$

where B (from Equation 1) is accounted for by the non‐protonated state of the hidden buffers, whatever their charge. To estimate C_tot_ and S_dp_, including also the contribution of hemoglobin, one should use the equation reported in the legend of Table 1, and the directly measured values of pH and of the buffer capacity of the blood sample. Alternatively, one can use the measured concentrations of hemoglobin, albumin, and phosphate (see Table 1), as in the so‐called Van Slyke equation (Lang & Zander, 2002; Siggaard‐Andersen & Fogh‐Andersen, 1995). Using K_a_ = 10^−7^ M, pH = 7.4, and the measured value of blood buffer capacity (minus the contribution of bicarbonate) I obtained the following values for human blood: C_tot_ = 77 mEq/L; S_dp_ = 79 mEq/L. These values are still underestimated, given that hemoglobin alone provides more than 80 mEq/L of His side chains; this is due to the approximations implicit in the estimate of C_tot_ and S_dp_ from an average value of K_a_. A more comprehensive model based on similar assumptions was developed by Kurtz and co‐workers (Nguyen et al., 2009).

The physico‐chemical model based on Equation 10 retains the concept of the constant value of the sum of deprotonated buffer components and uses the three independent and easily measurable parameters PCO_2_, pH, and the buffer capacity of blood (from which C_tot_ and S_dp_ are derived; notice that once these parameters have been calculated, they are independent of PCO_2_, whereas β is dependent on this parameter), is an excellent instrument for introducing students (and physicians) to the complexity of a mixture of two (or more) buffers, one of which is a dissolved gas (Table 2), and may also be used to simulate pathological conditions of the acid–base equilibrium in vivo (Table 3). This achievement is not to be underestimated: to rephrase a famous sentence of Nobel laureate Richard Feynman: “what I cannot calculate, I do not understand”.

**TABLE 3:** Simulation of some respiratory and metabolic pathophysiological conditions according to the modified model of Stewart.

	Healthy condition	Acute resp. acidosis	Chronic resp. acidosis	Acute resp. alkalosis	Chronic resp. alkalosis	Metabolic acidosis	Metabolic alkalosis
Non‐C buffer (C_tot_)	***77 mEq/L***	***77 mEq/L***	***77 mEq/L***	***77 mEq/L***	***77 mEq/L***	***77 mEq/L***	***77 mEq/L***
pH	***7.4***	***7.2***	***7.3***	***7.6***	***7.55***	***7.2***	***7.5***
PCO_2_	***38 mmHg***	***70***	***80***	***20***	***25***	***25***	***60***
[HCO_3_^−^]_plasma_	***23.5 mM***	***26.4 mM***	***38 mM***	***19 mM***	***21.1 mM***	***9.4 mM***	***45 mM***
*β*	*38 mEq/L*	*45.5 mEq/L*	*43.5 mEq/L*	*29.5 mEq/L*	*31.7 mEq/L*	*43.3 mEq/L*	*35.5 mEq/L*
S_dp_	*79 mEq/L*	*68 mEq/L*	*81 mEq/L*	*76 mEq/L*	*77 mEq/L*	*55 mEq/L*	*94 mEq/L*
pH_standard_	*7.4*	*7.27*	*7.42*	*7.37*	*7.37*	*7.1*	*7.55*
[HCO_3_^−^]_std_	*23.5 mM*	*17.8 mM*	*25.3 mM*	*22.4 mM*	*22.6 mM*	*11.9 mM*	*28 mM*
*β*_standard_	*38 mEq/L*	*42.3 mEq/L*	*37.3 mEq/L*	*39.2 mEq/L*	*39.1 mEq/L*	*45.8 mEq/L*	*32.4 mEq/L*
BE	0	−3 mEq/L	8.4 mEq/L	0.32 mEq/L	0.87 mEq/L	−16 mEq/L	19 mEq/L
**[HCO**_**3**_^**−**^ **]**_**optimal**_	**23.5 mM**	**–**	**40 ± 2 mM**	**–**	**18 ± 2 mM**	**–**	**–**
**PCO**_**2 optimal**_	**40 mmHg**	**–**	**–**	**–**	**–**	**22 ± 2 mmHg**	**55 ± 2 mmHg**

*Note*: Variables PCO_2_, [HCO_3_^−^]_plasma_, and pH were chosen in Arbus' diagram (bold italics); *β* for the healthy condition was taken from Ellison et al. (1958), and used to calculate C_tot_, assumed to be invariant (i.e., the concentrations of Hb, albumin and phosphate were assumed to be the same as in healthy subjects). Variables in italics (S_dp_, [HCO_3_^−^]_standard_ pH_standard_ and [HCO_3_^−^]_std_) were estimated by applying the equations described in the text. Base excess (BE) was calculated using Van Slyke equation (Lang & Zander, 2002); it refers to the standard parameters approach. Expected optimal compensation parameters (in bold) were calculated using the empirical equations of the physiological, bicarbonate‐based approach.

An interesting application of this model is the description of the arterio‐venous difference of total CO_2_. As already stated, the lung excretes 1.6–2 mmoles of CO_2_ per liter of blood as a response to a very small gradient of PCO_2_ of ≈5–7 mmHg. This extremely high efficiency is achieved thanks to the hidden buffers, and one can easily calculate that a lower concentration of these, or a lower value of S_dp_ would result in a much lower excretion of CO_2_ or would require a much higher gradient (see Table 2).

**FIGURE 3:**
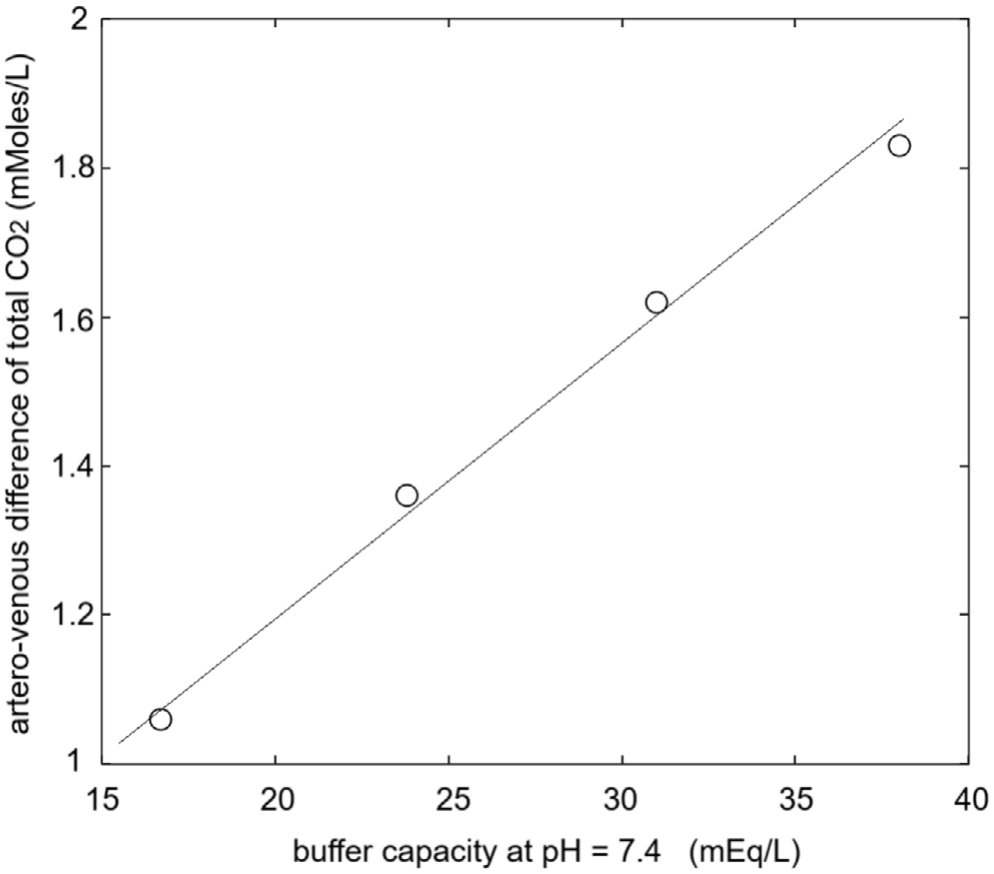
Simulated relationship between the buffer capacity of blood and the arterio‐venous difference in total CO_2_ at a constant ΔPCO_2_ (arterial PCO_2_ = 38 mmHg, venous PCO_2_ = 45 mmHg). The buffer capacity was decreased by decreasing C_tot_ and its contribution to S_dp_, as it would occur in severe anemias (see text).

One can go further and use Equation 10 to simulate the arterio‐venous difference of total CO_2_ for the physiological values of PCO_2_ as a function of the buffer capacity of the system that may change under pathological conditions (e.g., anemia). Figure 3 shows that the relationship between buffer capacity and arterio‐venous difference of total CO_2_ (ΔPCO_2_ values as in Table 2) is almost linear within the physiologically interesting range. Obviously, this analysis neglects other possible physiological adaptations (e.g., increased ΔPCO_2_).

Equation 10 can also be used to simulate some pathological conditions, even though under these conditions the estimates of C_tot_ and S_dp_ may become less reliable because a measured buffer capacity may not be available. Some examples are reported in Table 3.

Several more clinically oriented critical analyses of Stewart's theory have been presented (Androgué et al., 2009; Kurtz et al., 2008; Siggaard‐Andersen & Fogh‐Andersen, 1995), and in some cases, clear discrepancies between the SID and other acid–base parameters have been reported [e.g., (Kerbl‐Knapp et al., 2024)]. Indeed, the reason why one can sometimes, or even often, use this approach and obtain a sensible interpretation of clinical cases is that physiological mechanisms (e.g., renal) may couple strong ions (e.g., chloride) with buffer ions (e.g., bicarbonate) (Seifter, 2014; Story, 2004).

## 6 CLINICAL INTERPRETATION OF THE BLOOD GAS ANALYSIS: STANDARD PARAMETERS OR BICARBONATE‐BASED?

Two important problems requiring the attention of physicians are the discrimination between pathological and compensatory effects, and the quantitative evaluation of acid accumulation or loss. The school of respiration physiopathology from Copenhagen has been the most active in pursuing these formidable tasks via the use of the so‐called “standard parameters” (Severinghaus & Astrup, 1985). The procedure is as follows: after the relevant parameters of an arterial blood sample have been measured, the sample is equilibrated under standard conditions: PO_2_ = 100 mmHg, PCO_2_ = 40 mmHg, T = 37°C, and the relevant parameters are measured again, and termed the standard parameters (Jorgensen & Astrup, 1957; Siggaard‐Andersen et al., 1960). The idea behind this practice is that equilibration under standard conditions should remove the respiratory component of the observed disturbance and reveal the “pure” metabolic component, be it compensatory or pathological. Unfortunately, the added information is not easy to interpret and requires the judgment of a trained physician, because the procedure does not distinguish per se between compensatory and pathological non‐respiratory contributions.

The first standard parameter introduced into clinical use, with little success, was standard pH, advocated by none other than Karl Albert Hasselbalch in 1916 (Severinghaus & Astrup, 1985), but replaced by standard bicarbonate in 1957, the concentration of bicarbonate in a blood sample equilibrated under standard conditions (Jorgensen & Astrup, 1957), and successively by base excess (BE) (Siggaard‐Andersen et al., 1960). Base excess is defined as the amount of hydrochloric acid required to titrate the blood sample equilibrated under standard conditions to pH = 7.4; if the standard pH is lower than 7.4, the sample is titrated to pH = 7.4 with sodium hydroxide, and the parameter is called base deficit (or the patient is said to have a negative base excess). Base excess can be, and usually is, calculated from easier‐to‐measure parameters, using an equation developed by Siggaard‐Andersen and dedicated to Van Slyke (Lang & Zander, 2002; Siggaard‐Andersen & Fogh‐Andersen, 1995). Base excess measures the state of bicarbonate and the hidden buffers without any assumption on them being acidic or basic in nature, thus avoiding the clumsy assumptions that plague the concepts Singer and Hastings' BB^+^ or Stewart's SID. A positive BE is indicative of metabolic alkalinization of blood and is observed in compensated respiratory acidosis and/or in metabolic alkalosis; a negative BE is indicative of metabolic acidification of blood and is observed in compensated respiratory alkalosis and/or in metabolic acidosis (see Table 3).

The standard parameters approach was criticized by the Boston school of respiratory physiopathology, because titrating the blood sample in vitro negates the contribution of the buffer capacity provided by the tissue fluids, which are in equilibrium with blood. Indeed, significant contributions to the buffer capacity of the whole body in vivo derive from the ability of bone tissue to retain hydrogen ions (Lemann Jr. et al., 2003) and from the large CO_2_ stores in the tissues (Cherniack & Longobardo, 1970). The extracellular fluid has low buffer capacity, but a much higher volume of blood, thus its contribution in vivo is relevant. This led to the so‐called “great trans‐Atlantic debate” (Schwartz & Relman, 1963; Severinghaus, 1993). Siggaard‐Andersen solved the issue by suggesting a correction of the base excess parameter that he called the standard base excess (SBE), meant to mimic the average buffer capacity of blood plus extracellular fluid. To determine the SBE the physician should dilute the blood sample in its own plasma to a hemoglobin concentration of 5 g/dL and then titrate the sample as described above. The rationale of SBE goes beyond the diagnosis of the acid–base imbalance and bears implications for therapy, as it estimates the amount of acid lost or retained by the whole body of the patient.

Unfortunately, the standard parameters, while yielding an exact quantification of the acid–base imbalance, do not immediately tell us its cause, be it pathological or compensatory (Schwartz & Relman, 1963). One possible alternative is to record the values of the relevant parameters in patients suffering from a single disease, diagnosed with certainty: in this type of patient, one may assume that only one pathological and one compensatory component are present. The correlation between the relevant parameters can usually be approximated to a straight line, from which an empirical formula may be derived (Berend, 2018; Winters, 1965). A summary of the most commonly employed formulas is as follows:

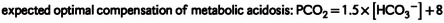




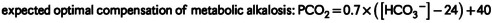




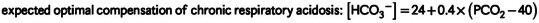




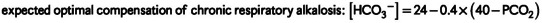

Allow an uncertainty of ±2 to the calculated values. Notice that the last two equations are identical but have been written differently to stress that the two compensations have opposite signs.

These formulas constitute useful rules of thumb to orient the physician: for example, if in a case of chronic metabolic acidosis, a PCO_2_ higher than expected is found, the physician may suspect an insufficient pulmonary compensation and hence a concomitant respiratory acidosis; if lower, hyperventilation may be present, leading to an overcompensation of the underlying disease. A similar reasoning is applied for the observed bicarbonate concentration in cases of respiratory acidosis.

## 7 CONCLUSIONS

The interpretation of the blood pH and its disturbances is a challenging physiological and clinical task that has been attacked from several different viewpoints, whose proponents have often been severely critical of each other. It seems evident that all these approaches can be reconciled with each other, and taken together they all contribute to a deeper understanding of this fascinating problem. The three main approaches examined in this work should not be considered alternative but complementary. Stewart's approach, after an extensive reformulation, provides an a priori conceptual framework that explains how the main variables of the system correlate to each other, but it is probably the least reliable in the clinical context (Androgué et al., 2009; Kurtz et al., 2008; Siggaard‐Andersen & Fogh‐Andersen, 1995). The physiological approach is probably the easiest to apply to the clinical context and produces useful rules of thumb for diagnosis, but it is purely empirical and does not contain an explanation of how the different parameters are correlated to each other. The base excess approach provides a more complete description of the clinical picture but requires extensive training to be mastered.

## 8 FUNDING INFORMATION

No funding was received.

## 9 ETHICS STATEMENT

This article does not involve experiments on humans or laboratory animals, nor does it contain sensible data.
